# Rubinstein-Taybi 2 associated to novel *EP300* mutations: deepening the clinical and genetic spectrum

**DOI:** 10.1186/s12881-018-0548-2

**Published:** 2018-03-05

**Authors:** María López, Alberto García-Oguiza, Judith Armstrong, Inmaculada García-Cobaleda, Sixto García-Miñaur, Fernando Santos-Simarro, Verónica Seidel, Elena Domínguez-Garrido

**Affiliations:** 1 Molecular Diagnostic Unit, Fundación Rioja Salud, Logroño, La Rioja Spain; 2 Department of Pediatrics, San Pedro Hospital, Logroño, Spain; 3 Servei de Medicina Genètica i Molecular, Institut de Recerca Pediàtrica and Department of Neurology Hospital Sant Joan de Déu (HSJD), CIBERER, Catalonia, Spain; 4 Unidad de Fertilidad y Diagnóstico Genético, Hospital Univ. Ntra. Sra. de La Candelaria, Santa Cruz de Tenerife, Spain; 5 Sección de Genética Clínica, INGEMM (Instituto de Genética Médica y Molecular), U753, CIBERER, Madrid, Spain; 6 Clinical Genetics, Department of Pediatrics, Hospital General Universitario Gregorio Marañón, Madrid, Spain

**Keywords:** RSTS, *EP300*-Rubinstein-Taybi, Broad thumbs, Intellectual disability, *EP300*-mutations, *EP300*-RSTS-phenotype, *EP300*, RSTS-2

## Abstract

**Background:**

Rubinstein-Taybi syndrome (RSTS) is a rare autosomal dominant neurodevelopmental disorder characterized by broad thumbs and halluces. RSTS is caused by mutations in *CREBBP* and in *EP300* genes in 50–60% and 8%, respectively. Up to now, 76 RSTS-*EP300* patients have been described. We present the clinical and molecular characterization of a cohort of RSTS patients carrying *EP300* mutations.

**Methods:**

Patients were selected from a cohort of 72 individuals suspected of RSTS after being negative in *CREBBP* study. MLPA and panel-based NGS *EP300* were performed.

**Results:**

Eight patients were found to carry *EP300* mutations. Phenotypic characteristics included: intellectual disability (generally mild), postnatal growth retardation, infant feeding problems, psychomotor and language delay and typical facial dysmorphisms (microcephaly, downslanting palpebral fissures, *columella* below the *alae nasi*, and prominent nose). Broad thumbs and/or halluces were common, but angulated thumbs were only found in two patients. We identified across the gene novel mutations, including large deletion, frameshift mutations, nonsense, missense and splicing alterations, confirming de novo origin in all but one (the mother, possibly underdiagnosed, has short and broad thumbs and had learning difficulties).

**Conclusions:**

The clinical evaluation of our patients corroborates that clinical features in *EP300* are less marked than in *CREBBP* patients although it is difficult to establish a genotype-phenotype correlation although. It is remarkable that these findings are observed in a RSTS-diagnosed cohort; some patients harbouring *EP300* mutations could present a different phenotype. Broadening the knowledge about *EP300*-RSTS phenotype may contribute to improve the management of patients and the counselling to the families.

## Background

Rubinstein-Taybi syndrome (RSTS; OMIM #180849, #613684) is a rare (1:125000) neurodevelopmental disorder. It affects equally males and females. This syndrome is characterized by a well-defined clinical features group including: variable degree of intellectual disability (ID), distinctive facial features (downslanting palpebral fissures, convex nasal bridge, *columella* below *alae nasi*, etc), skeletal abnormalities (broad and angulated thumbs and halluces, or duplication of distal phalanxes), growth retardation, microcephaly and behavioural problems. Broad and angulated thumbs and halluces are considered hallmarks in clinical diagnosis [1, 2].

The diagnosis of RSTS is essentially clinic based on frequent clinical characteristics such as: broad short thumbs and halluces, downslanting palpebral fissures, broad nasal bridge, hypotonia, gastrointestinal problems and recurrent infections, among others. Usually the diagnosis is made at an early stage, even at birth. However, there are some cases with milder features that lack diagnosis until adulthood [3, 4].

The first gene associated with RSTS is *CREBBP,* located on chromosome 16p13.3, that encodes a CREB-binding protein (CBP) [5]. Mutations in the gene *EP300* were detected in individuals clinically diagnosed of RSTS, setting up this gene as an alternative cause of RSTS [6]. *EP300* maps to 22q13.2 and encodes E1A-associated protein p300. CBP and p300 are ubiquitously expressed nuclear proteins, have intrinsic lysine acetyltransferase (KAT) activity and act as transcriptional coactivators in the regulation of gene expression mediating many of the same signalling pathways. They have been defined as “writers”. By acetylating histones, they loosen up the contact between histones and DNA, causing the relaxation of the chromatin and faciliting the access of transcription factors (TFs) and the basal transcriptional machinery to specific DNA sequences. RSTS is therefore classified as a disorder of the histone machinery. The fact that a defect in either *CREBBP* or *EP300* leads to the same syndrome might indicate that both histone acetyltransferases are targeted to an overlapping set of genes [7, 8]. 50–60% of RSTS cases are caused by mutations of the *CREBBP* gene, and by *EP300* gene mutations in around 8%. Thereby, in approximately one third of the RSTS patients the molecular cause of the syndrome remains unknown [9].

To date, about 230 causative mutations have been reported in *CREBBP*, in more than 200 patients. However, to the best of our knowledge, only 76 RSTS patients with *EP300* mutations have been described [10–14].

In this report we present the clinical and molecular characterization of a cohort of 8 RSTS patients carrying *EP300* mutations identified from a group of 72 RSTS patients. The description of more RSTS patients, and specifically *EP300*-cases may contribute to better understand the range of phenotypes providing clinical pointers that would improve earlier detection and diagnosis of these patients.

## Methods

### Patients

RSTS patients who underwent *EP300* analysis were selected from a cohort of 72 individuals with suspected diagnosis of RSTS after being negative in *CREBBP* study (Multiplex ligation-dependent probe amplification (MLPA) and next generation sequencing (NGS) of the entire gene.

Clinical data, samples and photographs were obtained after written informed consent. This work has been approved by the Committee for Ethics in Clinical Research in La Rioja (CEICLAR).

### Molecular analyses

Blood samples from probands and their parents, when possible (in two cases it was not possible), were collected in EDTA tubes. DNA was extracted using QIAamp DNA Mini Kit (QIAGEN) following the manufacture’s protocol. MLPA of *EP300* was performed (P333 Kit, MRC-Holland). If negative, NGS of *EP300* gene was carried out. Briefly, libraries encompassing exons and introns of *EP300* gene were prepared using the SureSelect^XT2^ Custom kit (Agilent) and sequenced to generate 150 bp single reads. The resulting reads were mapped to the human genome hg19 using BWA (version 0.7.1 2). Sequence variants were called using the Genome Analysis Toolkit (GATK) version 3.3 and called variants were annotated with Annovar. Pathogenicity of the detected variants was predicted by in silico analysis with bioinformatics tools such as Sorting Intolerant From Tolerant (SIFT), Mutation Taster, Polyphen-2, and Human Splicing Finder (HSF 3.0). ExAC browser of Broad Institute, 1000 Genomes database and dbSNP138, as well as, the Human Gene Mutation Database (HGMD), Leiden Open Variation Database (LOVD) and ClinVar databases were checked to assess the presence/absence of detected alterations in variations repositories.

All the pathogenic variants detected were corroborated by Sanger sequencing.

## Results

### Phenotype of EP300-RSTS patients

From the initial cohort of 72 patients, 8 of them harboured mutations in *EP300* gene, representing 11%. Phenotypic characteristics of these patients are summarized in Table 1 (for patients #47 and #57 there were not data available about some of these features since they were only 3 and 6 months old at the moment of the evaluation). The group of RSTS-*EP300* individuals included 5 males and 3 females aged from 3 months to 21 years old. Diagnosis was made at early stage in 2 cases, pediatric in 6 and adulthood in one (one of the probands´ mother) [4]. According to their phenotype, RSTS was clinically suspected in all these patients.

**Table 1:** Clinical features and genotype of *EP300* patients

Patient		#11^1^	#27^1^	#38	#42	#45^2^		#47	#57	#67
						DAUGHTER	MOTHER			
EP300 mutation	Nucleotide change	c.4954_4957dup	c.3728 + 5G > C	c.3163C > T	c.6627_6638del	c.7222_7223del		c.4511 T > G	DEL:Ex. 12–21	c.70_71del
	Predicted effect	p.(Cys1653Tyrfs*21)		p.(Arg1055*)	p.(Asn2209_Gln2213delinsLys)	p.(Gln2408Glufs*39)		p.(Phe1504Cys)		p.(Ser24Glyfs*14)
	Exon	30	In 21	17	31	31	31	28	12 to 21	1
	Number of reads (variant/total)	17/74	143/286	71/140	N.A. (SANGER SEQ)	238/486	219/485	282/581	N.A. (MLPA)	35/81
Age		18	16	21	9	9	42	< 1	< 1	10
Sex		M	M	M	M	F	F	M	F	F
Gestational problems	Preeclampsia	N.D.	mild hypertension		N	N		Y	N	N
	Prenatal growth retardation	N.D.	Y	N.D.	N	Y		N	N	N
	Other				N			high risk in triple screening, premature		
Growth delayed			N	N		Y	Y	N.D.	N.D.	Y
Intellectual disability	Mild		Y			Y	Y	N.D.	N.D.	Y
	Moderate	Y		Y				N.D.	N.D.	
	Severe				Y			N.D.	N.D.	
Pysochomotor delay		Y	Y	Y	Y	N	N	N.D.	N.D.	N
Language delay		N	Y	Y	Y	N	N	N.D.	N.D.	N
Behavioral problems	Anxiety	N	N	N	Y	mild	N	N.D.	N.D.	N
	Autism, autism-like	N	Y	stereotypes	Y	N	N	N.D.	N.D.	N
	Other		ADHD		hearing bizarries					ADHD
Typical facial dysmorphims	Arched eyebrows	N	N	Y	Y	N	N		N	
	Thick eyebrows	Y	Y	Y	N	N	N		N	Y
	Long eyelashes	Y	N	Y	Y	Y	Y			Y
	Microcephaly	Y	Y	Y	Y	Y	Y	Y	Y	Y
	Downslanting palpebral fissures	Y	Y	Y	Y	Y	N		N	Y
	Columella below the alae nasi	Y	Y	Y	Y	Y	Y	Y	±	Y
	Prominent nose	Y	Y	Y	Y	Y	Y	Y	N	N
	Narrow palate	Y	Y	Y	Y	Y	N		N	Y
	Narrow mouth	Y	Y	Y	N	Y	N		N	N
	Grimacing smile	N	N	Y	Y	N	N	N	Y	Y
	Posteriorly rotated ears	N	Y	N	N	Y	Y	N	N	Y
	Low set ears	N	Y	N	N	N	N	N	N	Y
	Other		micrognathia		large tongue			micrognathia, ocular hypertelorism		
Skeletal abnormalities	Broad thumbs	Y	N	Y	Y	Y	Y	Y	±	Y
	Angulated thumbs	Y	N	N	Y	N	N	N	N	N
	Broad halluces	Y	Y	Y	Y	N	N	N	±	N
	Scoliosis	N	N	N	N	N	N	N	N	N
	Other	delayed bone age	*Pextus excavatum*							
Skin abnormalities	Keloids	Y	N	N	heals badly	N	N	N	N	N
	Hirsutism	Y	N	Y	N	N	N	N	N	Y
Urinary tract anomalies		cryptorchidism	N	hydrocele	N	N	N	N	N	N
GI problems	Infant feeding problems	Y	Y	N	binge eating	N	N.D.	N	N	N
	Others								hypoketosic hypoglycemia	
Teeth malformations	Dental crowding	Y	Y	Y	N	N	N	N.D.	N.D.	Y
	Talon cusp	N	N	N	N	N	Y	N.D.	N.D.	N
	Malocclusion	N	Y	Y	N	N	N	N.D.	N.D.	N
	Other									
Eye anomalies	Strabismus	N	Y	Y	N	N	N	N.D.	N.D.	Y
	Coloboma	N	N	N	N	N	N	N.D.	N.D.	N
	Other		myopia		N	hypermetropia				hypermetropia

N.D.: no data available. ADHD: Attention Deficit Hyperactivity Disorder. ^1^Previously described by Fergelot et al. 2016. ^2^Case previously reported by Lopez et al. 2016

Only in six cases it was possible to retrieve information regarding the prenatal period: preeclampsia was found in one (case #47) and mild hypertension in other (#27); prenatal growth retardation in cases #27 and #45. Postnatal growth retardation was found in cases #67 and #45, as well as in her mother. Infant feeding problems were recorded in two cases (#11 and #27).

Psychomotor delay was observed in four cases (#11, #27, #38 and #42) and ID was present in all cases, being mild in 3/7, moderate in 4/7 and severe in 1/8. Language delay was present in three cases (#27, #38 and #42). Concerning behavioral problems, autism/autism-like was reported in three cases (#27, #42 and #67) and another one presented stereotypes.

**Fig. 1:**
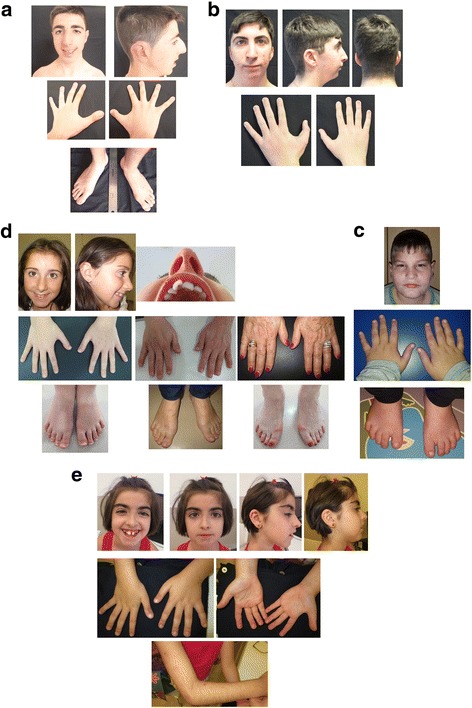
Photographs of face, hands and feet of *EP300* patients: #11 (**a**), #27 (**b**), #42 (**c**), #45 (**d**), #67 (**e**). For patient #45 photographs showing normal thumbs and halluces of the proband, short and broad but not angulated thumbs of her mother and her grandmother as well as detail of mother’s talon cusp at an upper incisor are shown

Microcephaly and typical facial dysmorphisms including downslanting palpebral fissures, *columella* below the *alae nasi a*nd prominent nose were found in almost all cases (Table 1, Fig. 1). Distinctive RSTS skeletal characteristics, broad thumbs and/or halluces were common, but angulated thumbs were only found in two patients (#11 and #42) (Table 1, Fig. 1).

Features previously reported like hirsutism or keloids were only present in patients #11 and #38. Other organ malformations such as dental crowding, talon cusp, strabismus, *Pectus excavatum* or cryptorchidism were also found (Table 1, Fig. 1).

### Genotype of EP300-RSTS patients

**Fig. 2:**
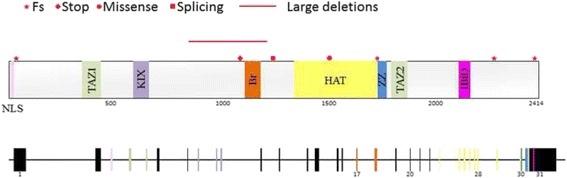
Schematic representation of p300 protein and *EP300* gene, including their functional and structural domains and their localization. Distribution of the variants found in *EP300* gene in our cohort are showed. Symbols represent the mutation types as indicated. The different domains of the p300 protein are represented with different colours, and the exon codifying these domains are showed in the same colour: pink (NLS, nuclear localization signal), green (TAZ1, transcriptional-adaptor zinc-finger domain 1), purple (KIX, kinase inducible domain of CREB interacting domain), orange (Br, bromodomain), yellow (HAT, histone acetyltransferase domain), blue (ZZ, bromodomain), green (TAZ2, transcriptional-adaptor zinc-finger domain 2), light pink (IBiD, IRF3-binding domain). Numbers indicate aminoacid position in the protein and the exon in the gene

Among our cohort of *EP300* patients we identified one large deletion, from exon12 to 21; 4 frameshift mutations (3 small deletions and 1 small insertion), 1 nonsense and 1 missense mutation and 1 intronic change with splicing alteration (Table 1, Fig. 2).

Large deletion was detected thorough MLPA analysis in patient #57. It displayed a multiexonic heterozygous deletion, from exon 12 to 21. It was not possible to confirm its de novo origin because of the absence of parents samples (Fig. 2).

Five novel heterozygous-inactivating mutations were identified: in patient #67 it was found a TC deletion (c.70_71del) which generates a premature stop codon, leading to an early truncated protein (p.(Ser24Glyfs*14)). This variant was not present in her parents, confirming its de novo origin. Patient #11 harboured a de novo small duplication (c.4954_4957dup) that gives place to a premature protein truncation (p.(Cys1653Tyrfs*21)). A transition from C to G was detected in patient #38, which converts an arginine triplet into a stop codon (c.3163C > T; p.(Arg1055*)); in this case there was not possibility of studying parents samples. In patient #27 an intronic variant was found, c.3728 + 5G > C. This change was predicted to alter the splicing removing the Natural Splice Site according to several predictors. RNA analysis corroborated that 22 nucleotides, belonging to the intron 21 sequence, were introduced between exons 21 and 22 leading to a premature stop codon. This variant was not present in parent samples.

Patient #42 has a de novo deletion in exon 31 (c.6627_6638del) which generates a deletion of five aminoacids, Asn-Gln-Phe-Gln-Gln, and a insertion of Lys (p.(Asn2209_Gln2213delinsLys)).

In the case of patient #47 a de novo missense variant located in exon 28 was identified (c.4511 T > G; p.(Phe1504Cys)). This point variation was predicted as deleterious, damaging, probably damaging and disease causing according to Provean, SIFT, Polyphen-2 and Mutation Taster, respectively. Although missense mutations in *EP300* have been hardly reported, the variant found in patient #47 (c.4511 T > G; p.(Phe1504Cys)) appear to be the causative of RSTS. It has been classified as pathogenic by different in silico analysis, have a Grantham score of 204.39 and a class C65 of align GVGD. Futhermore, this de novo variant is located in a functionally significant and conserved amino acid.

Analysis of the sample of patient #45 revealed a novel heterozygous frameshift mutation in exon 31 (c.7222_7223del; p.(Gln2408Glufs*39)). The deletion generates a frameshift that leads to loss of the original stop codon and results in a prolonged protein 31 aminoacids longer. However, the variant, considered to be pathogenic/likely pathogenic, according to ACMG interpretation, was present also in her mother, who also had learning difficulties and was found to have short and broad thumbs. These results were previously published [4].

All the variants detected were found in heterozygosity, as it is pointed out by the number of reads (Table 1). Moreover, all were confirmed by Sanger sequencing. To the best of our knowledge, these variants are novel (although two of them were previously described [14], Table 1), and are not included in ClinVar, HGMD or LOVD. They have been submitted to ClinVar (SCV266471, SCV000297724-SCV000297728, SCV000301482) and LOVD databases (variants #0000096186, #0000127944–0000127949, #0000130337). Furthermore, none of the detected variants was found in 100 healthy controls and are not present in 1000G, ExAC and dbSNP, pointing out that they are not common in population; with the exception of c.6627_6638del in patient #42 in which the role of this mutation is not clear.

## Discussion

In this report we present the genetic and clinical characterization of 8 new cases of RSTS associated to mutations in *EP300* gene. These results enlarge the number of *EP300*-RSTS cases to 84, broadening the knowledge about clinical presentation of these patients.

All the patients included in this study presented typical signs pointing to RSTS. The characteristics in *EP300* mutated patients are less marked than in *CREEBP* ones, in general. Even so, microcephaly and typical facial anomalies of RSTS were generally present in our cohort: downslanting palpebral fissures, *columella* below the *alae nasi*, and prominent nose; although other RSTS dysmorphic signs were only partially found among our patients (low set and rotated ears, grimacing smile, etc), supporting previously reported data (Table 2) [10, 12, 14].

**Table 2:** Comparison of typical features in *EP300*-patients found in literature

Feature	%		
	This work	Fergelot et al. 2016	Hamilton et al. 2016
Female/male	37.5/62.5	35/65	66/44
ID:			
Mild	37.5	62	56
Moderate	50	31	44
Severe	12.5	7	0
Broad thumbs	87.5	69	89
Angulated thumbs	25	2	ND
Microcephaly	100	87	67
Psychomotor delay	28.5	ND	89
Language delay	28.5	ND	100
Autism/Autistic behavior	28.5	25	33
Downslanting palpebral fissures	83	56	0
*Columella* below *alae nasi*	100	92	78

It should be noted that our work describes 8 cases, the paper by Fergelot et al. 52, and Hamilton et al. described 9 cases, 5 of them with no previous diagnosis of RSTS

Respecting skeletal malformations, 88% of *EP300*-individuals displayed broad thumbs and only two cases also angulated, confirming previous results that showed the uncommon presence angulated thumbs in *EP300*-RSTS patients (Table 2) [7, 14].

Milder RSTS features overall were recorded in this group, in comparison with the phenotype of *CREBB-*patients (prenatal growth retardation, delayed growth, infant feeding problems, hirsutism and keloids) were absent or detected in a low percentage. In line with literature, neurological affectation was generally mild, ID was generally mild and most of patients did not show psychomotor or speech delay. In addition, despite the recent description of high burden of behavioral difficulties in RSTS2 by Hamilton et al. [10], we have not detected enrichment in this problem among our cohort. In the same vein, even if several studies have set up an association between maternal pre-eclampsia and fetal *EP300*-RSTS only one of our cases registered this clinical fact (another one suffered mild hypertension). Nevertheless, the number of cases is not significative and these data could not be collected in all of them [7, 10–12, 14, 15].

Even if the number of cases in our study is not representative, it agrees in the most common type of variant found, small insertions and deletions that generate a frameshift. Our results subscribe with the last data reported by Spena et al. [9] who shows in a recent review that 53.5% of mutations in *EP300* are frameshift type, 25% nonsense, 14% large deletions, 3.6% missense and 3.6% splicing type (intronic).

Taking into account the location of mutations within *EP300*, it is important to point out that those found towards the 3′-end of the protein may result in milder phenotypes [3, 10, 15]. Furthermore, the number of mutations in exon 31 is lower, maybe because the phenotype in these patients is different and they are therefore not studied and lacked. In our study, patients #42 and #45 present mutations in this region. In the case of #45 this fact is corroborated, since the patient and her mother presented a mild phenotype with well preserved intelligence (in fact, the mother was not diagnosed before); however, patient #42 showed severe intellectual disability. Nevertheless, the role of this mutation is controversial, since it was present in ExAC, and the possibility of a second mutation separate to *EP300* making a significant contribution to his phenotype has not been entirely excluded.

It is remarkable that the only missense mutation found in this work was located in HAT domain. Although this kind of mutation is not commonly found as causative of RSTS (only a single missense mutation listed in LOVD), our variant is certainly pathogenic, because it is located in the HAT domain, it affects a highly conserved amino acid and in silico analysis predicted its pathogenicity. Accordingly, other authors found similar cases of apparently pathogenic missense mutations in *EP300* (Hamilton et al. 2016). In this regard, some authors described that probably missense mutations that affects HAT domain could lead to classical RSTS [14].

It has not been possible to establish a clear correlation between the genotype, type and location of mutations in *EP300*, with the phenotype, neither in our study, nor in the previous publications. This could be due to the low number of cases described and the wide spectrum of clinical features detected, emphasizing on the clinical variability of this syndrome. It is important to remark that our findings are observed in a RSTS-diagnosed cohort; some patients harbouring *EP300* mutations present a different phenotype as it has been demonstrated by the detection of *EP300*-mutated individuals through “hypothesis free” approaches (microarray or exome sequencing) without a previous clinical diagnosis of RSTS [10, 13, 16]. These cases without an initial RSTS diagnosis and “typical” *EP300*-phenotype plus other uncommon attributes, support the wide clinical spectrum of this syndrome. Possibly, *EP300* mutations may be more prevalent than we suppose, but the phenotype is different or overlaps with other syndromes, and as a result, molecular testing for RSTS is not offered.

The description of recurrent mutations in patients sharing the same defects and specific clinical signs as well as the description of other *EP300*-patients without RSTS diagnosis will help to set possible genotype-phenotype associations. Despite this fact, the clinical evaluation of our patients corroborates that clinical features in *EP300* are less marked than in *CREBBP* patients, being severe ID as well as angulation of thumbs and halluces, rare.

## Conclusions

In summary, it is difficult to establish a genotype-phenotype correlation, although alterations in HAT domain are proposed to cause classical RSTS and mutations not affecting this domain could explain the milder phenotypes. However, it is hampered by the low number of *EP300*-RSTS cases described worldwide and the clinical heterogeneity found, even among the few cases described with the same mutation. Broadening the knowledge about *EP300*-RSTS phenotype may contribute to improve the management of patients and the counselling to the families.

## Abbreviations

CBP: CREB-binding protein
CEICLAR: Committee for Ethics in Clinical Research in La Rioja
GATK: Genome Analysis Toolkit
HAT: Histone acetyltransferase
HGMD: Human Gene Mutation Database
ID: intellectual disability
KAT: lysine acetyltransferase
LOVD: Leiden Open Variation Database
MLPA: Multiplex ligation-dependent probe amplification
NGS: next generation sequencing
RSTS: Rubinstein-Taybi syndrome
SIFT: Sorting Intolerant From Tolerant
TFs: transcription factors
